# Transcriptomes analysis of *Aeromonas molluscorum* Av27 cells exposed to tributyltin (TBT): Unravelling the effects from the molecular level to the organism

**DOI:** 10.1016/j.marenvres.2015.06.017

**Published:** 2015-08

**Authors:** Andreia Cruz, Raquel Rodrigues, Miguel Pinheiro, Sónia Mendo

**Affiliations:** a Biology Department & CESAM, University of Aveiro, Campus de Santiago, 3810-193, Aveiro, Portugal; b Advanced Services Unit, Biocant – Biotechnology Innovation Center, 3060-325, Cantanhede, Portugal; c School of Medicine, University of St. Andrews, North Haugh, KY16 9TF, St. Andrews, UK

**Keywords:** Tributyltin, Quantitative real-time PCR, Pyoverdine, Dioxygenase, Acriflavine

## Abstract

*Aeromonas molluscorum* Av27 cells were exposed to 0, 5 and 50 μM of TBT and the respective transcriptomes were obtained by pyrosequencing. Gene Ontology revealed that exposure to 5 μM TBT results in a higher number of repressed genes in contrast with 50 μM of TBT, where the number of over-expressed genes is greater. At both TBT concentrations, higher variations in gene expression were found in the functional categories associated with enzymatic activities, transport/binding and oxidation-reduction. A number of proteins are affected by TBT, such as the acriflavin resistance protein, several transcription-related proteins, several Hsps, ABC transporters, CorA and ZntB and other outer membrane efflux proteins, all of these involved in cellular metabolic processes, important to maintain overall cell viability.

Using the STRING tool, several proteins with unknown function were related with others involved in degradation processes, such as the pyoverdine chromophore biosynthetic protein, that has been described as playing a role in the Sn–C cleavage of organotins.

This approach has allowed a better understanding of the molecular effects of exposure of bacterial cells to TBT. Furthermore it contributes to the knowledge of the functional genomic aspects of bacteria exposed to this pollutant. Furthermore, the transcriptomic data gathered, and now publically available, constitute a valuable resource for comparative genome analysis.

## Highlights

• RNA-Seq was used to study molecular effects of tributyltin on *Aeromonas molluscorum*.
• Gene expression was repressed at 5 μM of TBT and induced at 50 μM of TBT.
• Enzymatic activities, transport and oxidation-reduction were highly affected by TBT.
• Proteins involved in cell protection and resistance mechanisms were affected by TBT.
• Proteins with unknown function were related to degradation processes.

## 1 Introduction

Tributyltin (TBT) is a hydrophobic organic derivative of tin (Antizar-Ladislao, 2008), that possesses broad-spectrum biocidal properties. It has several applications, such as the production of PVC, agrochemicals, glass, textile, etc. (Antizar-Ladislao, 2008; Cruz et al., 2015). The most relevant application of TBT is its use as a component of antifouling paints (Pain and Cooney, 1998). In 2003, the European Union prohibited its usage on ships, and later, in 2008, TBT was banned by the International Maritime Organization (Antizar-Ladislao, 2008). Despite the widespread prohibition, and considering that it is a persistent organic pollutant, TBT is still causing several environmental problems (Chapman and Guillette, 2013) and negative effects in both prokaryotes and eukaryotes (Cooney and Wuertz, 1989a). In *Escherichia coli*, for instance, TBT affects growth, solute transport, biosynthesis of macromolecules and activity of transhydrogenases (Cruz et al., 2012; Singh and Singh, 1985; Singh, 1987). In eukaryotes, the most deleterious effect of TBT is imposex, which is the superimposition of male characters onto females (Smith, 1971). Due to its role as an endocrine disruptor, TBT has also been considered a xenobiotic obesogen (Grun et al., 2006).

Effects of TBT in prokaryotes have been reported which include: i) disturbance of the integrity of biological membranes, compromising their physiological functions (Alzieu et al., 1989; Cooney and Wuertz, 1989b; von Ballmoos et al., 2004); (ii) inhibition of the uptake of amino acids and of bacterial cell growth (Jude et al., 1996; Singh and Bragg, 1979b); (iii) inhibition of membrane-bound ATPase and energy-dependent pyridine dinucleotide transhydrogenase (Singh and Bragg, 1979a); (iv) disturbance of cell activity, due to alterations in the equilibrium and cell metabolism (Bossier and Verstraete, 1996; Cruz et al., 2012); and (v) inhibition of ATP synthetase and related enzymes (von Ballmoos et al., 2004).

Dubey et al. (2006) analysed the transcriptomes of *Pseudomonas aeruginosa* 25W using a microarray approach, and suggested that in this strain, TBT induces the expression of genes involved in cell protection and increased protein synthesis. The same authors observed that when the bacterium was exposed to 50 μM of TBT, minor effects were observed, whereas a higher concentration of TBT (500 μM) resulted in considerable changes in gene expression (6 genes were up-regulated and 75 were down-regulated). The most affected genes were those involved in the transcription and translation processes.

Despite the negative effects of TBT in bacteria, some species are resistant/tolerant to this compound (Cruz et al., 2007; Dubey et al., 2006; Fukagawa and Suzuki, 1993; Mimura et al., 2008; Sakultantimetha et al., 2009). Several mechanisms have been proposed to explain bacterial TBT resistance. These may be due to their ability: (i) to degrade TBT into dibutyltin (DBT) and monobutyltin (MBT) by dealkylation and demethylation processes (Barug, 1981; Clark et al., 1988; Pain and Cooney, 1998); (ii) to exclude TBT from the cell through efflux pumps or other membrane proteins (Jude et al., 2004); (iii) to metabolize TBT and use it as a carbon source (Kawai et al., 1998), and (iv) to bioaccumulate TBT using metallothionein-like proteins (Blair et al., 1982; Fukagawa et al., 1994).

Most of the available studies are focused on observations at the cellular level rather than at the molecular level. Therefore, in the present study a next generation sequencing approach was employed to better understand and clarify, at the molecular level, the effects of TBT in marine bacteria. *Aeromonas* species are intrinsically resistant to TBT (Cruz et al., 2008; Hernould et al., 2008) and are ubiquitous in marine environments. Thus *Aeromonas molluscorum* Av27 was used to conduct the transcriptomic analysis. This bacterium was isolated in an estuarine system and it is highly resistant to TBT (at least up to 3 mM). Moreover, Av27 degrades TBT into the less toxic compounds, DBT and MBT, when grown in Marine Broth (Cruz et al., 2007) and in natural sediment (Cruz et al., 2014). In addition, Av27 can be safely used to bioremediate TBT-polluted environments (Cruz et al., 2013).

The information concerning possible resistance mechanisms/degradation of TBT in bacteria are still scarce and inconclusive. The analysis of *A. molluscorum* Av27 transcriptomes contributes to this knowledge by providing relevant information on the molecular basis of these mechanisms. To the best of our knowledge, this is the first study using RNA-Seq approach to identify bacterial genes, whose expression is affected by exposure to TBT.

## 2 Materials and methods

### 2.1 Bacterial growth conditions

*A. molluscorum* Av27 cells (∼10^8^ cells L^−1^) were grown in Marine Broth (Difco) with or without tributyltin chloride (TBTCl) (97%) (Fluka), at 25 °C, the optimal growth temperature for this bacterium, 180 rpm, in the dark (to avoid TBT photo-degradation). Hereafter, TBTCl will be referred to as TBT.

Three replicates of three different conditions, were tested: (i) control, cells grown in media without TBT; (ii) cells exposed to 5 μM TBT, an environmentally relevant concentration (Antizar-Ladislao, 2008); and (iii) cells exposed to 50 μM TBT, which is a concentration that the strain is able to degrade (Cruz et al., 2007).

Cell growth was monitored by a change in optical density (OD) at 600 nm. At the exponential growth phase (OD_600 nm_ of 0.5) cells were pelleted and kept at −80 °C.

### 2.2 RNA extraction and purification

RNA was extracted using TRIzol Max Bacterial Isolation Kit (Invitrogen) according to manufacturer's instructions, and further purified with the Turbo DNA-free Kit (Ambion).

### 2.3 cDNA library construction and normalization

Total RNA (≈3 μg) was sent to Evrogen company (Russia), where double-stranded cDNA suitable for non-directional cloning was synthesized using the SMART approach (Zhu et al., 2001). A normalization step was performed, using a duplex-specific nuclease (DSN) normalization method (Zhulidov et al., 2004).

### 2.4 Roche 454 sequencing and data analysis

The transcriptomes, resulting from non-TBT-exposed and TBT-exposed conditions, were sequenced and annotated by Biocant (Portugal). Pyrosequencing was performed with 454 GS FLX Titanium. 454 reads were trimmed for removal of reads with less than 100 bp and low complex areas. Additionally, ribosomal, mitochondrial and chloroplast reads were removed. The remaining reads were assembled into contigs using 454 Newbler 2.6.

To identify the translations frames, the contigs were BLASTx against Swissprot using a threshold of e-value ≤10^−6^. An internal algorithm was used to translate the regions. The contigs without previous translation were run through FrameDP (Gouzy et al., 2009) to identify putative peptides; the remaining contigs without translation were run by ESTScan (Lottaz et al., 2003). The resulting putative proteins were annotated using the BLASTp against the nr@ncbi database. Functional annotation and Gene Ontology (GO) identification were obtained using the InterproScan (www.ebi.ac.uk/Tools/pfa/iprscan/).

To identify potential differential expression genes, the putative proteins were first clustered using a CD-Hit 454 application (Niu et al., 2010) (90% similarity) to eliminate redundant sequences. Then, contigs encoding non-redundant proteins were used as reference to map the reads; 454 Newbler Mapping 2.6 was used in the mapping process. These steps allowed the quantification of the number of reads that mapped in the references formed by the contigs. The application MyRNA (Langmead et al., 2010) was applied to obtain statistical evidence of the differential protein expression levels.

Sequencing raw data can be downloaded from Sequence Read Archive (SRA) through the BioProject PRJNA182603 accession number.

### 2.5 Identification of genes affected by TBT

The transcriptomes of *A*. *molluscorum* Av27 were analysed to identify genes affected by the presence of TBT.

Normalization of the libraries was performed to obtain an higher coverage of genes and to guarantee that large part of genes that are highly transcribed are not sequenced (Ekblom et al., 2012). This approach does not affect the differential expression gene results (Ekblom et al., 2012).

The expression ratio between TBT-exposed and non-exposed samples was calculated using the number of reads for each gene at each condition. The analysis involved the identification of over-expressed (expression ratio ≥ 2) and under-expressed (expression ratio < 0.5) genes, and the study of the respective GO. Only significant differences in gene expression (p-value ≤ 0.05) were considered to be regulated by TBT.

### 2.6 Validation of transcriptomes results by quantitative real-time PCR (qPCR)

Sixteen genes were selected (See Table S1, Supporting Information) based on their function and expression profile in the transcriptomes results. Their respective expression levels were determined by qPCR. To this end, Av27's cells were grown as described in the “Bacterial growth conditions” section. RNA was extracted and purified with the Rneasy Mini Kit (Qiagen), according to manufacturer's instructions and cDNA was synthesized using the SuperScript™ First-Strand Synthesis System for RT-PCR (Invitrogen).

To assess the gene expression levels, qPCR was performed in a CFX96™ real-time system (Bio-Rad), coupled with the C1000™ Thermal Cycler (Bio-Rad), using SoFast™ EvaGreen^®^ Supermix (Bio-Rad), and according to manufacturer's instructions. The thermal cycle profile was 30 s at 98 °C, followed by 50 cycles of 95 °C for 5 s and 60 °C for 15 s. Each reaction was followed by the analysis of a melting curve with a temperature range from 65 to 95 °C, with increments of 0.5 °C and 5 s/step to allow assessment of the reaction specificity. The primers used are shown in Table S1 (Supporting Information). The analysis included three biological and two technical replicates. A control sample (without the reverse transcriptase enzyme) and a non-template control (RNase-free water instead of cDNA) were also included. The gene expression level was determined by relative quantification and calculated with the ΔΔCt method (Biosystems, 2004), using *gyr*B and *rlm*L as internal controls (reference genes). Statistical analyses by one-way ANOVA (qbasePLUS, Biogazelle) were applied.

In qPCR, relative quantification does not provide the exact amount of the target gene, but allows comparison of gene expression between treated and untreated samples. Hence, the expression ratios between the treated samples (5 μM and 50 μM TBT) and the control sample (no TBT) were calculated by one-way ANOVA, followed by a Dunnett's test. Genes with expression ratio values ≥1 were considered to be over-expressed, while genes with ratios <1 were considered to be under-expressed. A p-value ≤0.05 was considered to be statistically significant.

### 2.7 Gene Ontology of proteins with unknown function

Some differentially expressed genes that presented homology with unknown proteins were analysed with STRING, version 9.1 (Franceschini et al., 2013). STRING allows predicting the gene functional network of the different proteins.

To assign GO and functional annotation, all 454 contig sequences from the control and exposed libraries were submitted to BLAST2GO (Conesa et al., 2005). This analysis was performed in order to explore the possible metabolic pathways in which respective proteins might be involved.

## 3 Results and discussion

### 3.1 Sequencing and assembly

Pyrosequencing of the three cDNA libraries generated a total of 247,550 reads with an average length of 321 bp. Specifically, 106,896 reads were obtained in the control library, which assembled into 1360 contigs. 60,378 reads were obtained in the library corresponding to the cells exposed to 5 μM TBT which were assembled into 1147 contigs. In the library corresponding to the cells exposed to 50 μM TBT, 80,276 reads were obtained, which were assembled into 1325 contigs (See Table S2, Supporting Information). These results reveal that the number of reads obtained in the control library is higher than the number of reads obtained in the libraries corresponding to the cells exposed to TBT. This difference is even more obvious at 5 μM TBT, and the results obtained from this library should be analysed taking this into consideration.

All the contigs were screened for open reading frames (ORFs) to predict the amino acid sequence of proteins resulting from these transcripts. The predicted ORFs were applied to InterproScan to identify their function, which also provided GO identification. A summary of the assembly and EST data obtained for each library is presented in Table S2 (Supporting Information).

### 3.2 Validation of transcriptomes analyses by qPCR

To validate the transcriptomes results, the expression level of 16 genes was determined by qPCR (See Table S1, Supporting Information). The genes were selected based on their different gene expression profile, and functional category. The analysis revealed that the relative expression level of these genes followed the same trend revealed by the relative expression obtained by the transcriptomes analysis (See Table S3, Supporting Information). Indeed, it was confirmed that the data retrieved reflect the bacterium transcriptional profile in response to TBT; thus, this information can be used to infer about genes that are affected by TBT exposure in bacteria.

### 3.3 Functional classification based on GO terms: identification of differential gene expression in response to TBT

The analysis of the transcriptomes allowed the identification of 1491 transcripts, 522 of which presented statistically significant differences (p-value < 0.05). After exposure to 5 μM TBT, 100 transcripts were over-expressed and 189 were under-expressed. As for 50 μM TBT, 247 transcripts were over-expressed and 223 were under-expressed. These results indicate that exposure to higher concentrations of TBT lead to higher changes in the gene expression profile.

**Fig. 1:**
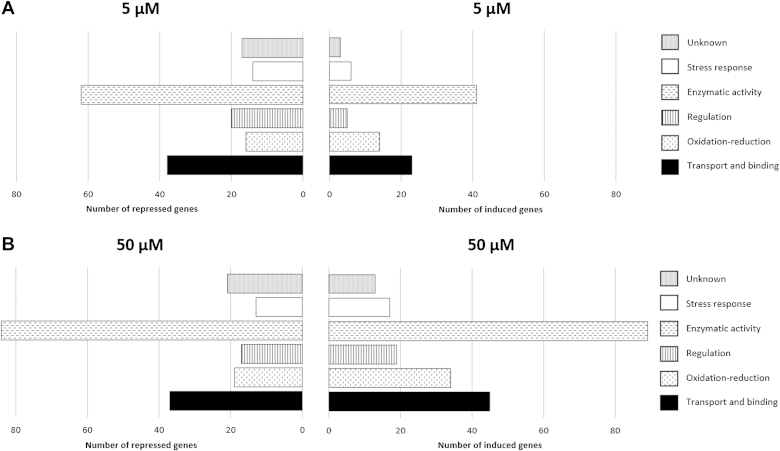
Functional categories of *A. molluscorum* Av27 over and under-expressed genes, following exposure to 5 μM (A) and 50 μM of TBT (B).

To further investigate specific processes, functions or cellular structures affected by TBT, GO annotations were performed. The information retrieved regarding GO associated with Biological Process, Cellular Component and Molecular Function identified in each condition (control, 5 μM and 50 μM TBT) is shown in Figure S1 (Supporting Information). In general, regarding Biological Processes, all of the tested conditions revealed most of the sequences assigning with “Metabolic Process” and “Cellular Process”. Furthermore, the matches of Cellular Component terms were prevalent within the “Cell”, “Cell Part” followed by the “Macromolecular Complex” category. Finally, for the Molecular Function GO the most dominant matches were found with the “Catalytic Activity” and “Binding” categories. The similarity of the results obtained between all the samples makes it difficult to draw conclusions. Thus, the number of over and under-expressed genes on each category was analysed, in comparison to the control samples (Fig. 1). This analysis revealed that exposure to 5 μM TBT results in a higher number of repressed genes in comparison with over-expressed ones, which could be related with energy saving and/or survival of the cell. Similarly, also to save energy, a repression of several genes was observed in *Lactococcus lactis* after infection by a bacteriophage (Fallico et al., 2011). Moreover, this result may also be related with the fact that fewer reads were obtained in the library corresponding to the cells exposed to 5 μM in comparison with the control library. At 50 μM of TBT, the number of over-expressed genes is higher than the repressed ones, which might be related with the activation of the defence mechanisms to withstand the stressful concentration. At both TBT concentrations, higher variation in both the over and under-expressed genes was found in the enzymatic activity functional category, followed by transport/binding and oxidation-reduction. These categories included genes that are mainly over-expressed at 50 μM (Table 1), suggesting their possible involvement to protect against TBT. It has been suggested that TBT can be exported from the cell through efflux pumps (Jude et al., 2004) and/or it can be degraded through dealkylation and demethylation (Barug, 1981), involving enzymes encoded by genes belonging to the above referred functional categories.

Table 1 lists some of the genes that are differentially expressed in the presence of TBT, among the different GO categories. Within the enzymatic activity category, proteins involved in DNA helix unwinding (IPR000606) and peptidoglucan synthesis (IPR000821) were highly over-expressed, suggesting that the DNA and the cell membranes are targets of TBT, as previously reported (Cruz et al., 2012).

Enzymes involved in oxidation–reduction processes are often involved in cell energy production. In the presence of TBT, the expression of these enzymes (e.g., IPR001327) was increased, ensuring basal cell activity.

Among the regulation category, a transcriptional regulator (IPR005119) of genes involved in virulence, metabolism, quorum sensing and motility was found over-expressed, suggesting an effect of TBT on the global fitness of the bacteria, which was also reported by Cruz et al. (2012).

Among the stress response category, different chaperonins and heat shock proteins showed increased expression levels when exposed to TBT. These proteins may help to maintain the correct folding of proteins that interact with TBT and whose activity may be inhibited (White et al., 1999).

Within the transport and binding category, a membrane protein (IPR000531) involved in iron uptake by active transport (Moeck and Coulton, 1998) was found over-expressed. Previously, other researchers (Inoue et al., 2003; Khanolkar et al., 2015) suggested that siderophore-like proteins might capture TBT. Thus, the results here reported support this hypothesis.

### 3.4 Identification of proteins potentially involved in TBT resistance mechanisms

Several proteins that were affected by TBT were identified. One of them is acriflavin resistance protein (IPR001036), which was under-expressed when cells were exposed to 5 μM of TBT and over-expressed when cells were exposed to 50 μM of TBT. This different behaviour can be due to the need of the cell to cope with a higher TBT concentration. When exposed to 50 μM of TBT, the cell may activate the expression of other proteins. Acriflavin is part of a multi-drug efflux system that possibly protects the cells against hydrophobic inhibitors (Kawamura-Sato et al., 1999), and it is also involved in the exclusion of antibiotics from the cell (Hobbs et al., 2012). Moreover, it is homologue to AheABC, an efflux pump associated with the intrinsic resistance to several compounds (Heroult et al., 2008). AheABC belongs to the resistance-nodulation-cell division (RND) family and showed to have substrate specificity and ability to export TBT in *Aeromonas hydrophila* ATCC 7966 (Hernould et al., 2008). Since TBT is a hydrophobic molecule it can be suggested that this protein might be also responsible for the efflux of TBT in Av27 strain.

Several transcription-related genes, such as, the translation elongation factor EFTu/EF1A, domain 2 (IPR004161) and the translation protein SH3-like (IPR008991), were found under-expressed upon exposure to TBT, demonstrating the effect of TBT in the transcription of several genes. Similar results were reported by Dubey et al. (2006) for the microarray analysis of *P. aeruginosa* 25W. In that study several transcription-related genes were found down-regulated after TBT exposure. A comparison of the results obtained in both studies suggests that TBT induces stress, which results in the inhibition of the transcription of these genes.

Also, several Hsps (Hsp70 (IPR001023), Hsp90 (IPR001404) and Hsp20 (IPR002068)) were found over-expressed in cells exposed to 50 μM TBT. These results are not surprising considering that TBT induces cellular stress. One possible role of these proteins might be on the repair of the damage caused by TBT, for instance, by helping on the re-folding of denaturated proteins, preventing protein aggregation and further degradation (Feder and Hofmann, 1999), and also by controlling the levels of some regulatory proteins (Gupta et al., 2010; Rohrwild et al., 1996). The results retrieved corroborate those of previous studies that showed that the expression of several Hsps was affected by the exposure to xenobiotics in both prokaryotes (Kaakoush et al., 2008) and eukaryotes (Yoshimi et al., 2002).

Other proteins allegedly involved in stress response caused by heavy metals were also found over-expressed in Av27 strain following TBT exposure. Among those are the ABC transporters (IPR001140), involved in ion export from the cell (Chang, 2003). These proteins showed higher expression levels in cells exposed to TBT. A similar expression profile was observed in the CorA and ZntB transporters (IPR002523), which mediate the transport of magnesium and zinc, respectively. In 1992, Suzuki et al. (1992) reported that *Vibrio* species were tolerant to both cadmium and TBTCl, and suggested a co-resistance to TBT and heavy metals. Hence, and accordingly, it can be suggested that these transporters, besides being involved in Av27 resistance to some heavy metals (mercury, copper, zinc and cadmium) (Cruz et al., 2007), might also be able to transport TBT.

Finally, the transcriptome results also revealed that, in the presence of TBT, the expression of an outer membrane efflux protein (IPR003423) was up-regulated. This protein is associated with the export of several substrates in Gram negative bacteria (Fralick, 1996). Thus, it appears that in *A. molluscorum* Av27 this protein has the function of exporting TBT.

### 3.5 Analysis of proteins with unknown function that are potentially involved in TBT uptake and degradation

Several unknown proteins presented alterations in the gene expression profile in cells exposed to TBT. STRING analysis revealed that some of those proteins are related to others found in the genome of *A. hydrophila* ATCC 7966, namely proteins involved in degradation processes. According to STRING analysis, the protein with unknown function DUF494 (IPR007456) showed to be close to the LysM domain-containing protein (AHA_0259), which in turn, is directly related with the pyoverdine chromophore biosynthetic protein PvcA (AHA_3282) and pyoverdine chromophore biosynthetic protein PvcB (AHA_3281). Pyoverdine is a siderophore that may play an important role in the Sn–C cleavage of organotins, in both the metal-free and metal-complexed states, in *Pseudomonas chlororaphis* (Inoue et al., 2003). Another unknown protein identified by the GO analysis was recognized as Pirin, N-terminal (AHA_3978; IPR003829). The gene encoding this protein maps close to MarR family transcriptional regulator (AHA_1539) that is close to the homogentisate 1,2-dioxygenase (AHA_2662) of *A. hydrophila* ATCC 7966. Homogentisate 1,2-dioxygenase has been described to be involved in TBT degradation in microalgae, via successive dealkylations (Lee et al., 1989; Tsang et al., 1999). Another protein with unknown function, DUF465 (IPR007420), is located in the vicinity of chorismate synthase (aroC) and of amonabactin (AHA_2479) in the ATCC 7966 strain's genome. Amonabactin is a siderophore that is involved in a mechanism of iron acquisition in *A. hydrophila* (Barghouthi et al., 1989).

Additionally, all 454 contig sequences from the control and exposed libraries were submitted to BLAST2GO to detect other genes potentially associated with TBT exposure. Accordingly, a synthase (IPR004561, AHA_2479) which was found over-expressed at 5 μM TBT seems to be involved in the biosynthesis of siderophores, and according to STRING is related to the enterobactin synthase subunit E (AHA_2478). Enterobactin is a siderophore involved in the acquisition of iron in *E. coli*. These siderophores are produced by many bacteria and are organic chelators with very high specific affinity for iron (III) (Braun and Hantke, 2011; Upritchard et al., 2011).

Several other proteins were also identified in Av27's transcriptomes that take part in different metabolic pathways associated with the degradation of compounds, such as fatty acids, drugs, xenobiotics, geraniol, naphthalene, toluene, xylene, dioxin and caprolactam. The proteins that are associated with a higher number of metabolic pathways were dehydrogenases with the enzymes commission (EC) number of 1.1.1.1, 1.2.1.10, 1.1.1.35 and 1.2.1.5. The first two dehydrogenases (EC. 1.1.1.1 and EC.1.2.1.10) are under-expressed in both transcriptomes (5 and 50 μM), and the remaining (EC.1.1.1.35 and EC.1.2.1.5) were found to be over-expressed only in the 50 μM TBT transcriptome. It has been reported that in several microorganisms, dehydrogenases are involved in xenobiotic phase I biotransformations (Oppermann and Maser, 2000; Tan, 1999). These results indicate that proteins involved in the sequestration and biodegradation of xenobiotic compounds are up-regulated in Av27 cells exposed to high TBT concentrations.

## 4 Conclusion

The present study intended to contribute to a better understanding of the molecular mechanisms concerned with the exposure of marine bacteria to the toxic compound TBT. Indeed, transcriptomes analysis of Av27 identified several proteins whose expression is affected by exposure to this toxicant. In general, it can be concluded that exposure to TBT at a concentration of 5 μM induced the expression of a number of proteins required to cell survival, more directly related to obtaining energy required to ensure cell survival. In contrast, when the cells are exposed to 50 μM of TBT a number of genes are activated that are more related with defence (e.g. resistance, degradation, stress response) mechanisms. Accordingly, the most affected genes belong to the functional categories involved in enzymatic activities, followed by transport/binding and oxidation-reduction. Several transcription-related genes, transporters and genes involved in defence and degradation mechanisms were identified.

The genome of *A. molluscorum* Av27 has not yet been sequenced, therefore the transcriptomic data herein presented, and publically available, gives key information regarding the molecular basis of the effects of the exposure to TBT in marine bacteria. In addition, it constitutes a valuable resource for further comparative genome analysis. Furthermore, the data retrieved will be the basis for further research that will help elucidate and clarify the TBT resistance/degradation mechanisms in bacteria which, in turn, is important for environment bio-restoration of contaminated sites.

## Figures and Tables

**Table 1:**
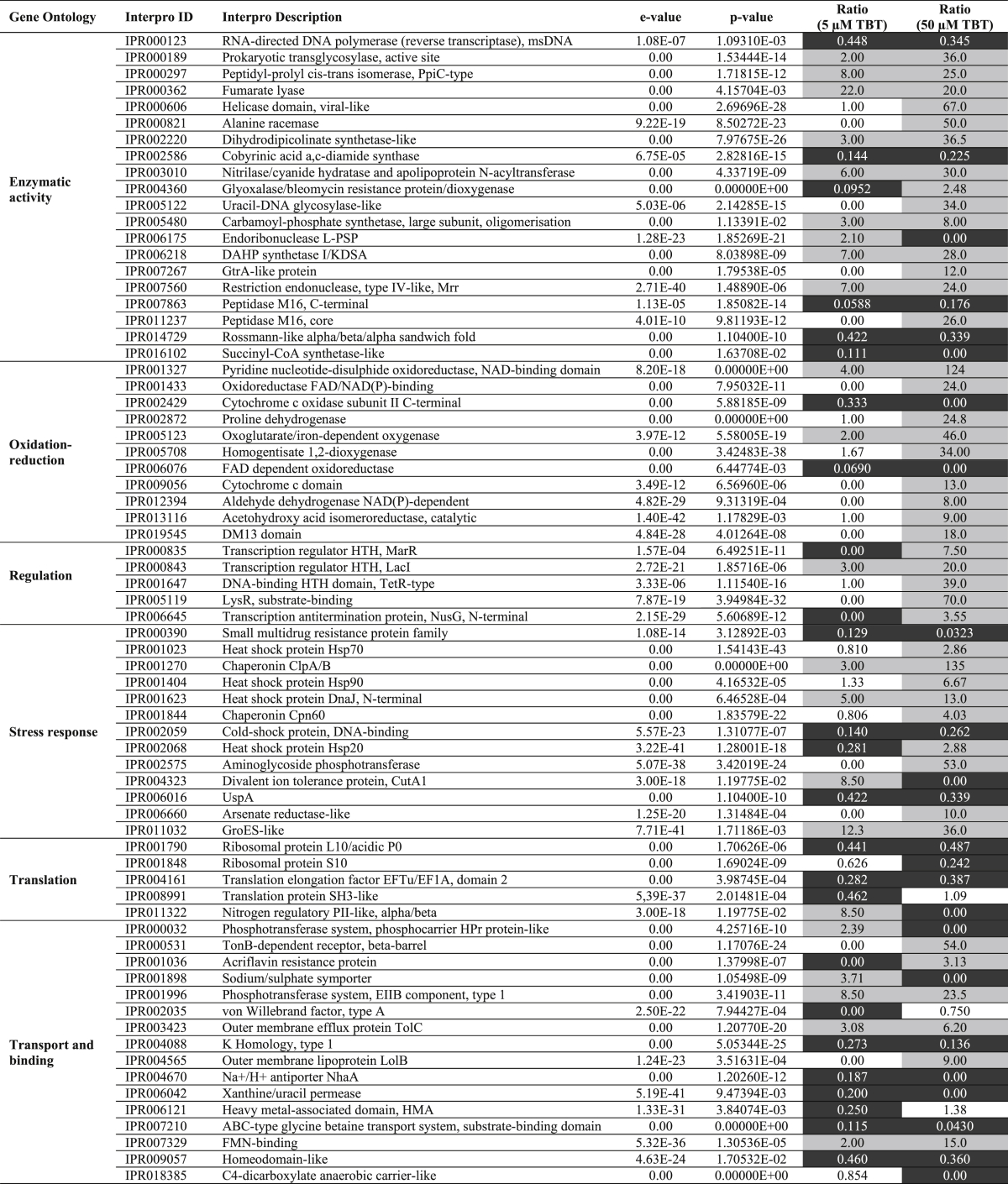

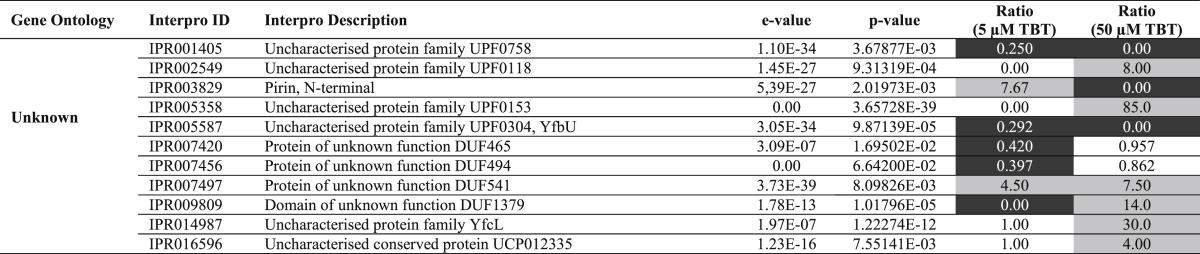
Summary of selected over-expressed and under-expressed genes in *A. molluscorum* Av27, following TBT exposure. Grey background: over-expressed genes (expression ratio ≥ 2), black background: under-expressed genes (expression ratio < 0.5).

## Appendix A Supplementary data

SI presents on Table S1 the Forward and reverse primer sequences used in qPCR analyses, on Table S2, a summary of assembly and EST data from *A. molluscorum* Av27 transcriptomes sequencing, on Table S3, a comparison of qPCR results and transcriptome analysis and on Fig. S1, the Gene Ontology (GO) results.
