# Supplementary material for: Transcriptomes analysis of Aeromonas molluscorum Av27 cells exposed to tributyltin (TBT): Unravelling the effects from the molecular level to the organism
Source: Mar Environ Res. 2015 Aug;109:132–9. doi: 10.1016/j.marenvres.2015.06.017 (PMC4541717; doi:10.1016/j.marenvres.2015.06.017)
Supplement: Supplementary file 1 [file mmc1.docx]

**Supplementary Material**

**Table S1.** Forward and reverse primer sequences used in qPCR analyses**.**

|  | **Gene** | **Forward primer (5’-3’)** | **Reverse primer (5’-3’)** |
| --- | --- | --- | --- |
| **Reference genes** | *gyr*B | TCCTCAACTCTGGCGTTTCT | GGGGAATGTTGTTGGTGAAG |
|  | *rlm*L | GAGCGCACTGGTGTCATCTA | GCGCATTCTGAGAGAGATCC |
| **Target genes** | B224_000468 | TGATGGTCACCTTCTTGCTG | GTACCGACCAGGGTGAAGAA |
|  | *sug*E | ATGCCCTGGATATTGCTGCTC | GGGTGAAACCTTGGGTGTATTTG |
|  | AHA_0734 | GAGGTGAGTGACGAGCTCAA | ATAGAGTGCAGTCGGGGTGA |
|  | AHA_0021 | GCCAGTTCTCCCTGACCATC | GGACATGATGACCCCCTGAT |
|  | *gro*S | TCATCATTAAGCGCATCGAA | GCCGTAACCTTCGTTGAAGA |
|  | B224_000472 | CAcGAGCTCGATCCATACAA | ATTCCGGATACACGAACAGC |
|  | IYQ_23030 | AAATGGCCAAGATGTCGTTC | TTGTGATGGACGAAACGGTA |
|  | *tol*C | TGTCAGTCATGGTCCTGCTC | TTGGCCAGGTAGTTGAGTCC |
|  | *cut*A | GACCTCATCAGCGAGCAACT | TGATGATGAGCTGGATCTCG |
|  | ASA_1926 | AGCCAACACCATCAATCCTC | GTGTTTGTGACGGGCTACCT |
|  | *nha*A | ACGATCTGGGCGTCATTATC | ACCCGACCAACATGTAGAGG |
|  | AHA_2405 | GCCAGAAGGACATCAAGGAG | GTACTGCTCCACCCAGGAGA |
|  | *hmgA* | GAGTTGCTGTTCATCCACGA | CTATCATTGGTCGCCTCGAT |
|  | AHA_3374 | ACATAGCGCAGCTCAAGGAT | ACTTCCAGCCAGACCACATC |
|  | AHA_1498 | GCCTTACACAACGAGCTGGA | TCAGATCCCTCAACTCAGGAC T |
|  | IYQ_07906 | GGAAGACAAGGTCCATCTGC | GGTTGGGATAGGCGTAGACA |

**Table S2.** Summary of assembly and EST data from *A. molluscorum* Av27 transcriptomes sequencing: control and TBT exposed libraries.

|  | **Control library** | **5 μM TBT library** | **50 μM TBT library** |  |
| --- | --- | --- | --- | --- |
| **Number of reads** | 106,896 | 60,378 | 80,276 |  |
| **Total number of bases** | 34,656,300 | 18,312,500 | 26,942,400 |  |
| **Average read length after trimming (bp)** | 324 | 303 | 335 |  |
| **Number of contigs** | 1,360 | 1,147 | 1,325 |  |
| **Average contig length (bp)** | 1,056 | 878 | 982 |  |
| **Range of contig length (bp)** | [77..16,243] | [141..13,769] | [133..13,973] |  |
| **Number of contigs with >2 reads** | 1,360 | 1,147 | 1,325 |  |
| **Peptides with E-value < 1e^-6^ (a)** | 1,549 | 1,181 | 1,429 |  |
| **Remaining peptides with frameDP (b)** | 336 | 226 | 306 |  |
| **Remaining peptides with ESTscan (c)** | 143 | 148 | 169 |  |
| **Total number of peptides (a+b+c)** | | 2,028 | 1,555 | 1,904 |
| **Amino acid sequence assigned to GO terms** | | 1,252 | 984 | 1,168 |
| **Amino acid sequence assigned InterPro terms** | 1,576 | 1,184 | 1,415 |  |
| **Amino acid sequence not assigned InterPro terms (from blastx E<1e^-6^)** | 53 | 30 | 57 |  |
| **Amino acid sequence not assigned InterPro terms (from FrameDP)** | 259 | 199 | 273 |  |
| **Amino acid sequence not assigned InterPro terms (from ESTScan)** | 140 | 142 | 159 |  |

**Table S3.** Comparison of qPCR results and transcriptome analysis: p-value and expression ratios (nº reads in the presence of TBT/nº reads in the absence of TBT) obtained for each target gene selected for validation. Grey background: over-expressed genes; black background: under-expressed genes.

|  |  |  | **Transcriptome analysis** | | | **qPCR results** | | |
| --- | --- | --- | --- | --- | --- | --- | --- | --- |
| **Interpro ID** | **Interpro description** | **Gene name** | **p-value** | **Ratio**  **(5 μM)** | **Ratio**  **(50 μM)** | **p-value** | **Ratio**  **(5 μM)** | **Ratio**  **(50 μM)** |
| IPR000298 | Cytochrome c oxidase, subunit III | B224_000468* | 9.115E-03 | 0.182 | 0.909 | 0.213 | 0.813 | 0.607 |
| IPR000390 | Small multidrug resistance protein family - SugE | *sugE* | 3.129E-03 | 0.129 | 0.032 | 0.153 | 0.681 | 0.476 |
| IPR000835 | Transcription regulator HTH, MarR | AHA_0734** | 6.493E-11 | 0.000 | 7.500 | 0.273 | 0.173 | 1.845 |
| IPR001036 | Acriflavin resistance protein | AHA_0021** | 1.380E-07 | 0.000 | 3.125 | 0.793 | 0.902 | 1.076 |
| IPR011032 | GroES-like | *groS* | 1.712E-03 | 12.33 | 36.00 | 0.525 | 1.485 | 1.128 |
| IPR002429 | Cytochrome c oxidase subunit II C-terminal | B224_000472* | 5.882E-09 | 0.333 | 0.000 | 0.468 | 0.808 | 0.696 |
| IPR002586 | Cobyrinic acid a,c-diamide synthase | IYQ_23030* | 2.828E-15 | 0.144 | 0.225 | 0.088 | 0.234 | 0.374 |
| IPR003423 | Outer membrane efflux protein TolC | *tolC* | 1.208E-20 | 3.080 | 6.200 | 0.606 | 1.045 | 2.454 |
| IPR004323 | Divalent ion tolerance protein, CutA1 | *cutA* | 1.198E-02 | 8.500 | 0.000 | 0.139 | 1.039 | 0.505 |
| IPR004360 | Glyoxalase/bleomycin resistance protein/dioxygenase | ASA_1926** | 0.000E+00 | 0.095 | 2.476 | 0.196 | 0.566 | 1.646 |
| IPR004670 | Na+/H+ antiporter NhaA | *nhaA* | 1.203E-12 | 0.186 | 0.000 | 0.206 | 0.852 | 0.692 |
| IPR005123 | Oxoglutarate/iron-dependent oxygenase | AHA_2405** | 5.580E-19 | 2.000 | 46.00 | 0.192 | 1.004 | 3.020 |
| IPR005708 | Homogentisate 1,2-dioxygenase | *hmgA* | 3.425E-38 | 1.667 | 34.00 | 0.110 | 2.481 | 2.835 |
| IPR007210 | ABC-type glycine betaine transport system | AHA_3374** | 0.000E+00 | 0.115 | 0.043 | 0.399 | 0.473 | 0.705 |
| IPR007420 | Protein of unknown function DUF465 | AHA_1498** | 1.695E-02 | 0.420 | 0.957 | 2.689E-02 | 0.897 | 0.278 |
| IPR007863 | Peptidase M16, C-terminal | IYQ_07906* | 1.850E-14 | 0.059 | 0.032 | 0.375 | 0.841 | 0.849 |

*: ORF name

**: Ordered locus name

**Fig. S1**. Gene Ontology (GO). Percentage of GO annotations for *A. molluscorum* Av27 sequences associated with Biological Process (A), Cellular Component (B) and Molecular Function (C).

**
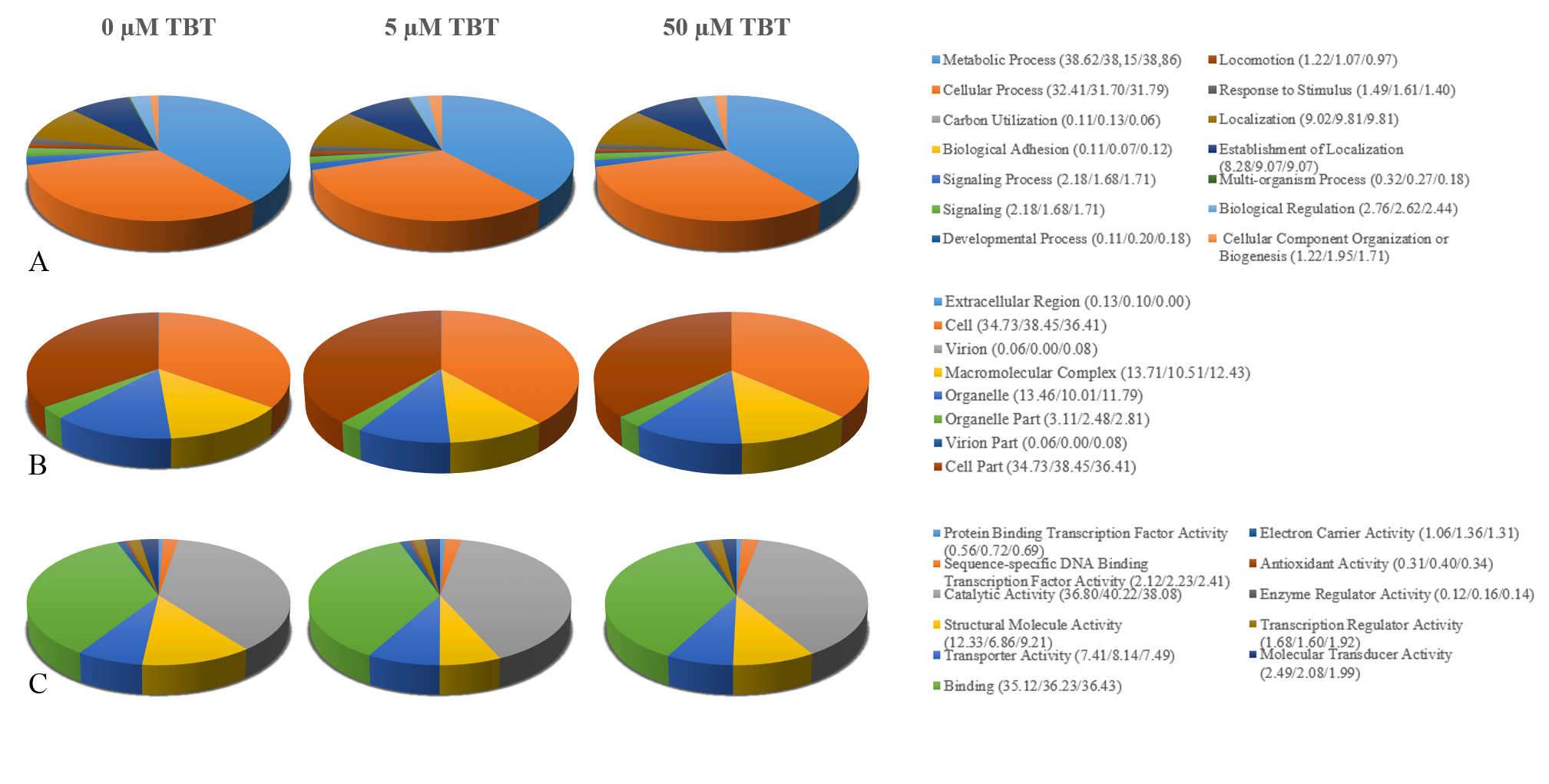
**
